# Comparison Between a Self-Administered and Supervised Version of a Web-Based Cognitive Test Battery: Results From the NutriNet-Santé Cohort Study

**DOI:** 10.2196/jmir.4862

**Published:** 2016-04-05

**Authors:** Karen E Assmann, Marion Bailet, Amandine C Lecoffre, Pilar Galan, Serge Hercberg, Hélène Amieva, Emmanuelle Kesse-Guyot

**Affiliations:** ^1^ Université Paris 13, Equipe de Recherche en Epidémiologie Nutritionnelle (EREN) Centre d'Epidémiologie et Statistiques Sorbonne Paris Cité Inserm (U1153), Inra (U1125), Cnam, COMUE Sorbonne Paris Cité Bobigny France; ^2^ Département de Santé Publique Hôpital Avicenne Bobigny France; ^3^ Inserm U897 Université Bordeaux Bordeaux France

**Keywords:** cognition, executive function, internet, cohort studies

## Abstract

**Background:**

Dementia is a major public health problem, and repeated cognitive data from large epidemiological studies could help to develop efficient measures of early prevention. Data collection by self-administered online tools could drastically reduce the logistical and financial burden of such large-scale investigations. In this context, it is important to obtain data concerning the comparability of such new online tools with traditional, supervised modes of cognitive assessment.

**Objective:**

Our objective was to compare self-administration of the Web-based NutriNet-Santé cognitive test battery (NutriCog) with administration by a neuropsychologist.

**Methods:**

The test battery included four tests, measuring, among others aspects, psychomotor speed, attention, executive function, episodic memory, working memory, and associative memory. Both versions of the cognitive battery were completed by 189 volunteers (either self-administered version first, n=99, or supervised version first, n=90). Subjects also completed a satisfaction questionnaire. Concordance was assessed by Spearman correlation.

**Results:**

Agreement between both versions varied according to the investigated cognitive task and outcome variable. Spearman correlations ranged between .42 and .73. Moreover, a majority of participants responded that they “absolutely” or “rather” agreed that the duration of the self-administered battery was acceptable (184/185, 99.5%), that the tasks were amusing (162/185, 87.6%), that the instructions were sufficiently detailed (168/185; 90.8%) and understandable (164/185, 88.7%), and that they had overall enjoyed the test battery (182/185, 98.4%).

**Conclusions:**

The self-administered version of the Web-based NutriCog cognitive test battery provided similar information as the supervised version. Thus, integrating repeated cognitive evaluations into large cohorts via the implementation of self-administered online versions of traditional test batteries appears to be feasible.

## Introduction

More than one third of the population in more developed world regions will be aged 60 years and older by the year 2050, according to estimates published in a United Nations report [1]. This population aging is taking place worldwide and is associated with a significant increase in the burden of age-related cognitive decline [2]. According to the 2009 World Alzheimer Report [3], the number of dementia cases is estimated to double every 20 years. Given the major social and economic consequences of dementia, age-related cognitive decline is one of the key public health problems of our time.

Current evidence consistently indicates that Alzheimer’s disease diagnoses are preceded by a very long pre-dementia phase [4-8]. Moreover, various studies suggest that this phase begins relatively early in life, indicating that studies on determinants of cognitive aging should focus on midlife factors [9-15]. Hence, longitudinal studies with available data on midlife risk factors and with repeated cognitive data are well adapted for the investigation of potential targets for early preventive measures [11]. Yet, the high logistical and personnel-related costs of traditional neuropsychological evaluations (ie, neuropsychological interviews or paper-and-pencil tests with supervision by trained personnel [16]) present major feasibility problems for epidemiological studies with large sample sizes. Thus, there has been a growing interest in the development of computerized cognitive evaluations as these allow for standardized, repeated procedures, systematic scoring, and automated saving or data management [17,18].

Two recent reviews of the literature have counted a total of 13 different computerized cognitive test batteries that are adapted to the context of age-related cognitive impairment or dementia [17,18]. Of these, only four are fully self-administered: the Computer-Administered Neuropsychological Screen for Mild Cognitive Impairment (CANS-MCI) battery, the Central Nervous System Vital Signs (CNS Vital Signs) battery, the MicroCog: Assessment of Cognitive Functioning (MicroCog) battery, and the Computer Assessment of Mild Cognitive Impairment (CAMCI) battery. One further battery, the Cambridge Neuropsychological Test Automated Battery (CANTAB) was described as “largely independent of verbal instructions.” Such fully self-administered batteries that do not rely on examiners to explain instructions or to supervise the completion of tasks are of interest for multiple reasons. Logistical and personnel costs can be further reduced, effort and time investment for participants are lower (since testing can be done at home), and an integration of cognitive evaluations into large-scale epidemiological studies is possible. However, it is important to assess the concordance of such a fully self-administered mode of administration with a “supervised” mode of administration. A supervised mode of administration is characterized by the presence of a trained examiner who gives test instructions, supervises the completion of the battery, and is available to help in the case of comprehension problems.

The objective of this study was to compare these two types of administration (fully self-administered versus supervised) with respect to a cognitive test battery (ie, NutriCog) that was specifically developed for the French NutriNet-Santé cohort.

## Methods

### Choice of the Cognitive Tasks to Include in the NutriCog Battery

The selection of the cognitive tasks included in our battery was based on a literature review of available computerized neuropsychological batteries [17,18]. Three important considerations guided our choices. First, the tasks had to be feasible in the context of an entirely Web-based battery (in relation to connection speed limitations, the use of mouse and keyboard instead of stylus pens, etc). Second, given our objective to study long-term age-related cognitive decline starting from midlife, the tasks had to reflect cognitive processes that have been shown to show slight functional changes in early aging (ie, episodic memory, attention/working memory, and highly integrated executive functions) [16,19]. Third, the selected tasks had to be derived from robust, previously validated neuropsychological paradigms. The tasks that were finally chosen to be part of our battery are described in Table 1. Briefly, the first task (“Click”) consists of connecting numbers (in ascending order) as fast as possible, the second task (“Maze”) consists of discovering a labyrinth route while respecting a certain number of rules, the third task (“Cards”) is a matching-to-sample task presented as a card game, and the fourth task (“Marbles”) consists of memorizing and recalling the color pattern of several marbles with a specific location on the screen. The specific realizations of all tasks except the Click-task are randomly generated at the beginning of each session and were thus not identical across the two modes of administration (fully self-administered/supervised). For example, participants were confronted with distinct labyrinth routes for each of the two completions of the test battery (fully self-administered/supervised).

**Table 1:** Description of the cognitive test battery, NutriNet-Santé Study.

Task name	Cognitive processes involved	Task description	Outcome variables
Click	Visual-motor coordination, psychomotor speed, selective attention	Numbers (1-5) are connected in ascending order (as fast as possible). A set of three consecutive trials is completed. The task is similar to part A of the Trail Making Test^a^.	Mean time taken (seconds)
Maze (Maze A, Maze B)	Episodic memory, procedural memory, working memory, visuospatial attention, executive function (planning, inhibition, mental flexibility)	A labyrinth path is discovered by the participant, who has to respect several different rules. First, a set of three consecutive trials is completed. Next, after performing the Cards task, the Marbles task, and a supplemental unrelated task (with a duration of a few minutes), the participant is presented with a final (fourth) trial. The task was adapted from the Groton Maze Learning test^b^.	Total time taken (seconds); number of total clicks (n); number of total errors (n)—These variables are calculated separately for the initial 3 trials (Maze A) and the final trial (Maze B)
Cards	Working memory, executive function (inhibition, mental flexibility), sustained attention	Matching-to-sample (one-back type) task: cards are continuously presented to the participant, who must decide whether or not a presented card is the same as the one shown just before.	([1/ (incorrect answers+1000)] /time) * 100,000
Marbles	Visual memory, episodic memory, associative memory	The participant is presented with a central marble and several colored peripheral marbles. First, the color patterns of each marble have to be memorized. Second, the colors of the peripheral marbles disappear and the central marble continuously takes on different colors, and the participant has to click on the peripheral marble corresponding to the color shown.	([1/ (incorrect answers+1000)] /time) * 100,000

^a^Details on the Trail Making Test have been published elsewhere [16].

^b^Details on the Groton Maze Learning Test have been published elsewhere [20].

### Development of an Operational Version of the NutriCog Battery

In order to obtain our final Web-based instrument, the NutriCog battery, the following working steps were undertaken: (1) adaptation of the task instructions to a self-administered computerized framework, (2) prototype development, (3) pilot testing, and (4) revision of the prototype. Pilot testing consisted of assessing comprehensibility of the instructions, timing, and potential technical glitches. These pilot tests have been conducted in our research institute, within a sample of individuals with varying age, education level, and sex.

Lessons that we have learned during the development of the operational version of our battery include the importance of constructive exchanges within an interdisciplinary team of neuropsychologists, epidemiologists, and computer scientists in order to find solutions that are relevant in terms of neuropsychological paradigms, the planned epidemiological investigations, and feasibility in terms of software development. Moreover, multiple rounds of pilot testing within samples of individuals with diversified characteristics were necessary to identify software errors and comprehensibility problems, and to calibrate the display times, for example, messages during the Maze task or cards during the Cards task.

### Design of the Comparison Study

In order to compare the self-administered mode of the NutriCog Web-based cognitive battery to a supervised mode of this same battery, we conducted a comparison study in a subsample of the NutriNet-Santé cohort. Each participant of this subsample was asked to perform the test battery twice (ie, in both the self-administered mode and the supervised mode), with an intermission of about 2 months (mean 73.2, SD 17.2 days) in between the two realizations. In order to account for learning effects that have to be expected when repeatedly administering cognitive tests [16], we randomized participants into two study groups, differing in the order of administration of the test battery version. The respective study groups were named SA-SU (self-administered version first, supervised version second) and SU-SA (supervised version first, self-administered version second). The NutriCog test battery was available on the NutriNet-Santé personal Internet page of each participant. The volunteers were asked to use at least 12-inch monitors for optimal visualization of the tests, to use a mouse, and to be in a quiet place without any disruption.

Prior to administering the battery of cognitive tests, the participants were asked to complete a short questionnaire assessing their current mood. At the end of each round of assessment (ie, self-administered version and supervised version), participants were presented with a process evaluation questionnaire in order to provide feedback on the test battery. The expected time to complete the battery in full was 20-25 minutes (15-20 minutes for the cognitive tests and 5 minutes for the questionnaires). Finally, after the participants had completed both versions of the test battery, they were presented with a satisfaction questionnaire, designed to collect information on the acceptability of the duration of each version of the battery, the difficulty of the tasks, the presentation and comprehensibility of the instructions, and the overall appreciation of the test battery.

### Administration of the Two Versions of the Test Battery

The self-administered version was completed by the volunteers while alone, following the instructions given on the webpage. The supervised version was completed with assistance by a trained neuropsychologist, who made a home-visit appointment with each volunteer. The instructions were given orally by the neuropsychologist, who was also available to answer any upcoming questions concerning the instructions. All administrations of the supervised version of the battery were realized by the same neuropsychologist. There were no other differences between the two assessment rounds.

### Selection of Participants for the Comparison Study

**Figure 1:**
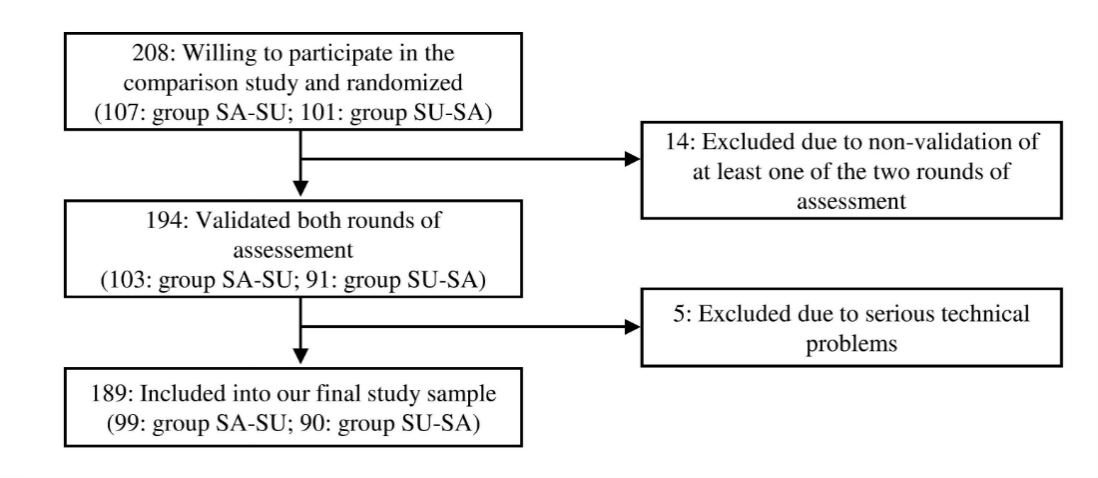
Selection of participants and attribution to the study groups SA-SU (self-administered version first, supervised version second) and SU-SA (self-administered version first, supervised version second).

The selection process for our study sample is presented in Figure 1. We selected a subsample of 1416 participants of the NutriNet-Santé cohort, via a stratified randomization procedure, using sex, age group (<50 years, ≥50 years), and educational level (≤2 years of higher education, >2 years of higher education) as stratification variables. Among those subjects who agreed to participate, we selected 208 individuals with varying sex, age, and educational level (our pre-defined objective was to obtain a final sample of approximately 200 participants) and randomly attributed them to the two study groups. Of these 208 subjects, 14 were excluded because they did not validate both versions of the test battery, and 5 individuals were excluded because serious technical (ie, computer-related) problems had occurred as they completed the battery. Thus, our final study sample consisted of 189 participants (group SA-SU: n=99; group SU-SA: n=90).

### Statistical Analyses

#### Creation of Composite Variables

For the Cards and Marbles tasks, composite variables relating the number of incorrect answers to the time taken were created, as both the quality of the responses and the rapidity of task completion are important for the evaluation of performances on these tasks. The following formula was applied in order to relate incorrect answers to time while obtaining readable (ie, not too small) values and improving the normality of the distribution: ([1/(incorrect answers+1000)] /time) *100,000. For these composite variables, higher scores indicate higher performances, while for all other variables, lower scores indicate higher performances.

### Descriptive Analysis

Participant characteristics were presented as n and % or p50 and p25, p75 (ie, 50^th^and 25^th^, 75^th^percentile) and differences across study groups were tested via chi-square tests and (nonparametric) Wilcoxon rank-sum tests, respectively. In order to detect potential learning effects, median performances on the first administration and on the second administration of the battery were tabulated and compared via nonparametric paired tests (Wilcoxon signed-rank tests). Furthermore, we tabulated median performances according to test battery version (self-administered, supervised) as p50 and p25, p75. Finally, Spearman correlations between the different cognitive outcomes variables were tabulated.

### Concordance Analysis

Concordance between the two versions of the test battery was estimated via Spearman correlation coefficients (presented with their *P* values). We decided to use Spearman correlations as our main statistical indicators in order to facilitate a comparison of our results with other methodological articles on computerized cognitive test batteries, which mostly report Pearson or Spearman correlations [18]. Spearman correlations were more adapted to our data than Pearson correlations as the cognitive outcome variables were not normally distributed.

Spearman correlations were presented for the whole study sample and stratified by age (<50 years, ≥50 years), educational level (≤2 years of post–high school education, >2 years of post–high school education), sex, and Web experience (Web novice, Web expert). Participants reporting that they were “inexperienced” or “beginners” in terms of Internet knowledge were designated as “Web novices” and participants report “advanced” or “expert” Internet knowledge were designated as “Web experts.”

Finally, participants’ responses to the satisfaction questionnaire were tabulated as n and %. All analyses were conducted with SAS, version 9.3, and all statistical tests were two-sided with a significance level of .05.

## Results

Our study sample had a median age of 57 years and 61.9% of the participants were women. Participant characteristics according to study group are presented in Table 2. Individuals in the SA-SU group were younger than participants in the SU-SA group and more frequently reported an advanced or expert level in Internet knowledge. In terms of gender, educational level, occupational category, and type of Internet connection, no significant differences between groups were observed.

Table 3 presents performances on the different cognitive tasks according to time of administration of the cognitive battery (first administration versus second administration). Except for the time taken on the “Click” task and for the “Marbles” task composite variable, the performances on the cognitive tasks were systematically better for the second administration.

For illustrative purposes, performances on cognitive tests according to version of the test battery (self-administrated, supervised) are presented in Multimedia Appendix 1. Multimedia Appendix 2 shows Spearman correlations among the different cognitive test variables for the self-administered version of the test battery.

Table 4 shows Spearman correlation coefficients as measures of concordance between cognitive performances according to version. Spearman correlations ranged between .42 and .73. The lowest correlations were observed for the outcome variables “mean number of clicks” and “mean number of total errors” of the Maze task (Maze A and Maze B): here, Spearman correlation coefficients ranged between .42 and .43.

Multimedia Appendix 3 shows Spearman correlation coefficients stratified by age group, educational level, sex, and Web experience. The coefficients ranged from .21-.80 and tended to be highest among participants who were higher educated and who had better Internet knowledge. However, these trends were not consistent across all outcome variables.

Tables 5 and 6 present the responses of participants to the satisfaction questionnaire. Notably, a majority of participants responded that they “absolutely” or “rather” agreed that the duration of the self-administered battery was acceptable (184/185, 99.5%), that the tasks were amusing (162/185, 87.6%), that the instructions were sufficiently detailed (168/185, 90.8%) and understandable (164/185, 88.7%), and that they had globally enjoyed the test battery (182/185, 98.4%). On the other hand, 81.1% (150/185) of participants reported to have preferred the supervised version of the self-administered version.

**Table 2:** Participant characteristics (N=189).

		Self-administered version first (n=99)		Supervised version first (n=90)		*P* ^b^
		n or p50^a^	% or p25; p75^a^	n or p50^a^	% or p25; p75^a^	
Age		55.0	40.0; 65.0	59.0	51.0; 67.0	.02
**Gender**						.27
	Male	34	34.3	38	42.2	
	Female	65	65.7	52	57.8	
**Education level**						.74
	<2 yrs post–high school education	34	34.3	33	36.7	
	≥2 yrs post–high school education	65	65.7	57	63.3	
**Occupational category**						.50
	Unemployed	13	13.1	7	7.8	
	Employee	8	8.1	10	11.1	
	Intermediate profession	12	12.1	8	8.9	
	Managerial staff^c^	38	38.4	32	35.6	
	Retired	28	28.3	33	36.7	
**Self-evaluated Web knowledge** ^**d,e**^						.049
	Web novice	6	6.1	13	14.9	
	Web expert	92	93.9	74	85.1	
**Type of connection** ^d^						.94
	<512k	9	9.2	9	10.3	
	≥512 and <1024k	18	18.4	16	18.4	
	≥1024k	48	49.0	39	44.8	
	Do not know	23	23.5	23	26.4	

^a^ Values for age are not n and %, but median and 25^th^; 75^th^percentile.

^b^ *P* value for the difference between both administration order groups, from Wilcoxon rank-sum nonparametric tests for age and chi-square tests for other variables.

^c^Or intellectual profession.

^d^185 subjects (98 for the self-administered first and 87 for the supervised version first) returned the satisfaction questionnaire, thereby providing this information.

^e^Novice: “inexperienced” or “beginner” level; expert: “advanced” or “expert” level.

**Table 3:** Performance on cognitive tests: first versus second administration (N=189)^a^.

Test^b^	Variable	First administration		Second administration		*P* ^c^
		p50	p25; p75	p50	p25; p75	
Click	Time in seconds, mean	4.29	3.64; 5.49	4.32	3.68; 5.36	.70
**Maze A**						
	Time in seconds, mean	118.66	86.95; 168.67	101.16	79.28; 129.76	<.001
	Clicks, mean n	59.00	53.00; 70.33	54.00	48.33; 60.67	<.001
	Total errors, mean n	17.33	13.33; 25.00	14.00	10.67; 18.00	<.001
**Maze B**						
	Time in seconds	85.35	62.87; 112.64	72.11	51.72; 92.99	<.001
	Clicks, n	48.00	42.00; 55.00	44.00	37.00; 51.00	<.001
	Total errors, n	10.00	7.00; 15.00	8.00	4.00; 12.00	<.001
Cards	Composite variable^d^	1.26	1.09; 1.36	1.28	1.15; 1.40	<.001
Marbles	Composite variable^d^	2.21	1.48; 3.31	2.40	1.77; 3.33	.18

^a^Objective of this comparison: identification of potential learning effects. Lower scores indicate better performances, except for the Cards and Marbles composite variables, where higher scores indicate better performances.

^b^Maze A: Sum of the initial three rounds of the Maze task. Maze B: Final (fourth) round of the Maze task.

^c^Wilcoxon signed-rank test (nonparametric paired test).

^d^([1/(incorrect answers+1000) ] /time) * 100,000.

**Table 4:** Concordance of cognitive tests performances according to version (N=189)^a^.

Test^b^	Variable	Spearman correlation	
		*r*	*P*
Click	Time in seconds, mean	.73	<.001
**Maze A**			
	Time in seconds, mean	.57	<.001
	Clicks, mean n	.43	<.001
	Total errors, mean n	.43	<.001
**Maze B**			
	Time in seconds	.53	<.001
	Clicks, n	.43	<.001
	Total errors, n	.42	<.001
Cards	Composite variable^c^	.64	<.001
Marbles	Composite variable^c^	.51	<.001

^a^Self-administered version versus supervised version.

^b^Maze A: Sum of the initial three rounds of the Maze task. Maze B: Final (fourth) round of the Maze task.

^c^([1/ (incorrect answers+1000) ] /time) * 100,000.

**Table 5:** Participant satisfaction with the two versions of the cognitive test battery, questions concerning the self-administered version (N=185)^a^_._

Questions		n	%
**The duration is acceptable**			
	I absolutely agree	145	78.4
	I rather agree	39	21.1
	I rather don’t agree	1	0.5
	I absolutely don’t agree	0	0.0
**The exercises can be rapidly finished**			
	I absolutely agree	64	34.6
	I rather agree	112	60.5
	I rather don’t agree	8	4.3
	I absolutely don’t agree	1	0.5
**The exercises are too simple**			
	I absolutely agree	4	2.2
	I rather agree	89	48.1
	I rather don’t agree	87	47.0
	I absolutely don’t agree	5	2.7
**The exercises are amusing**			
	I absolutely agree	51	27.6
	I rather agree	111	60.0
	I rather don’t agree	23	12.4
	I absolutely don’t agree	0	0.0
**The instructions are sufficiently detailed**			
	I absolutely agree	80	43.2
	I rather agree	88	47.6
	I rather don’t agree	17	9.2
	I absolutely don’t agree	0	0.0
**The instructions are understandable**			
	I absolutely agree	69	37.3
	I rather agree	95	51.4
	I rather don’t agree	20	10.8
	I absolutely don’t agree	1	0.5
**Overall, I have appreciated the test battery**			
	I absolutely agree	83	44.9
	I rather agree	99	53.5
	I rather don’t agree	3	1.6
	I absolutely don’t agree	0	0.0
**I have had problems visualizing the graphics**			
	I absolutely agree	2	1.1
	I rather agree	7	3.8
	I rather don’t agree	49	26.5
	I absolutely don’t agree	127	68.7
**If I was proposed to repeat the test battery, I would agree**			
	I absolutely agree	155	83.8
	I rather agree	30	16.2
	I rather don’t agree	0	0.0
	I absolutely don’t agree	0	0.0

^a^Only 185 out of the 189 participants of the study completed the satisfaction questionnaire.

**Table 6:** Participant satisfaction with the two versions of the cognitive test battery, questions concerning the supervised version, and comparison of the two versions (N=185)^a^.

Questions			n	%
**Questions concerning the supervised version**				
	**The duration is acceptable**			
		I absolutely agree	157	84.9
		I rather agree	28	15.1
		I rather don’t agree	0	0.0
		I absolutely don’t agree	0	0.0
	**I have appreciated completing the tests with a professional**			
		I absolutely agree	128	69.2
		I rather agree	56	30.3
		I rather don’t agree	0	0.0
		I absolutely don’t agree	1	0.5
**Comparison of the two versions**				
	**Overall, which of the two versions have you preferred?**			
		The Web version	35	18.9
		The supervised version	150	81.1

^a^Only 185 out of the 189 participants of the study completed the satisfaction questionnaire.

## Discussion

### Principal Findings

The aim of this comparison study was to assess the concordance of the fully self-administered mode of the Web-based NutriCog test battery with a supervised mode of the same battery, characterized by the presence of a trained neuropsychologist. Nine different outcome variables were evaluated to measure the performance of participants on four different tasks (Click, Maze, Cards, and Marbles). Spearman coefficients for the correlation between the two modes of administrations, in terms of cognitive test performances, ranged between .42 and .73. Correlation coefficients were lower for the Maze task (Mazes A and B) than for the other tasks. This could indicate potential problems concerning the comprehensibility of the Maze task instructions in the absence of a supervisor. However, based on suggestions made by the participants and by the neuropsychologist who was present during the supervised version of the test battery, these instructions have been slightly revised (after the comparison study was completed), in order to enhance understanding.

The observed correlation coefficients varied according to educational level and Web knowledge. Correlations tended to be higher among higher educated participants and among those with higher Internet knowledge. However, as can be expected in the context of an entirely Web-based study, there were only very few individuals who reported being “inexperienced” or having a “beginner level” with respect to Web knowledge.

Overall, in the context of this comparison study, very high values of concordance could not be expected due to multiple circumstances. First, we observed a clear learning effect, with test results that were almost systematically better at the second administration of the test battery, independently of the mode of administration. If such learning effects were of similar magnitude for the whole study sample, this would not affect the calculated Spearman correlations. On the other hand, differential learning effects would have lowered the observed concordance values in our sample. Further, cognitive performances are subject to a rather large amount of intra-individual variation [21].

To the best of our knowledge, no other study has yet investigated the concordance of a fully self-administered version of a cognitive test battery with a supervised version of the same test battery. However, the context of our study is similar to studies investigating test-retest reliability (ie, the correlation of performances on a first and a second administration of the same test battery), which have been conducted for multiple computerized cognitive batteries. A systematic review of the literature by Tierney et al identified 11 computerized cognitive test batteries [18], of which nine had information on the test-retest reliability for each subtest. The respective ranges of (Pearson or Spearman) correlation coefficients were .30-.74 (CAMCI) [22], .65-.88 (CANS-MCI) [23], .56-.90 (CNS Vital Signs) [24], .53-.93 (Cognitive Drug Research Computerized Assessment System for Dementia, COGDRAS-D) [25], .23-.79 (Cogstate) [26], .68-.80 (CSI) [27], .59-.98 (short form of the MicroCog battery) [28], and .40-.84 (Mindstreams Mild Impairment Battery) [29]. For the CANTAB battery, intraclass correlation coefficients were reported instead of Pearson or Spearman correlations, with a range of .09-.86 [30]. Of note, in our study, intraclass correlation coefficients (which were calculated after applying transformations to improve normality) ranged from .36-.65 (data not shown).

In this study, lower correlation coefficients than for such test-retest investigations had to be expected as we compared two different modes of administration. Although very high values of ≥.9 for specific subtests (as observed for the CNS Vital Signs, COGDRAS-D, and Microcog batteries) were not obtained in this investigation, the range of correlation coefficients found in our study (.42-.73) is roughly comparable to the ranges found in other studies.

Another element that supports the use of our Web-based cognitive battery in a fully self-administered mode is the fact that a majority (88.7%) of participants evaluated the test instructions as sufficiently detailed and understandable. As stated above, in order to further improve the comprehensibility of the test battery, the instructions concerning the Maze task have been slightly modified by taking into account the suggestions of participants of this comparison study. Besides, 81.1% of participants preferred the supervised version of the battery to the self-administered version. However, this is probably largely due to the fact that social interaction with a health professional was perceived as a more pleasant situation than completing the test battery alone.

### Strengths and Limitations

A certain number of limitations to this study have to be considered. First, the observed learning effect between the first and second administration of the test battery is difficult to separate from differences in cognitive performances that are related to the mode of administration. Second, this comparison study aimed only to compare the fully self-administered mode of the battery to a supervised mode of administration. Data on the ability of the battery to accurately discriminate normal cognitive function from impaired cognitive function are not available. An important strength of our study is its originality, as to the best of our knowledge, no other study has yet compared full self-administration to supervised administration of cognitive batteries. Moreover, the study was conducted within a rather large sample of 189 participants of varying age, sex, and educational level. Finally, the concordance between the two modes of administrations was assessed for the whole population as well as for specific subgroups.

### Conclusions

The concordance of the self-administered version and the supervised version of the Web-based NutriCog cognitive test battery was roughly similar to that observed for test-retest investigations of other test batteries. This indicates that these two different modes of administration provide similar information. In large epidemiological studies like the NutriNet-Santé cohort, the objective of cognitive evaluations is not to provide data with high validity in a clinical context, but to measure cognitive performances in a rapid and simple manner, with sufficient quality to permit valid conclusions on the population level. Given the drastic reduction of the logistical and financial burden that can be obtained by using fully self-administered tools, Web-based cognitive test batteries such as NutriCog provide interesting alternatives to supervised tools in the context of large cohort studies.

## Appendix

Multimedia Appendix 1 Performance on cognitive tests according to version.

Multimedia Appendix 2 Spearman correlations between the different cognitive test variables for the self-administered version of the cognitive test battery NutriCog.

Multimedia Appendix 3 Concordance of cognitive tests performances according to version: Spearman correlation coefficients, stratified by participant characteristics.

## Abbreviations

CAMCI: Computer Assessment of Mild Cognitive Impairment
CANS-MCI: Computer-Administered Neuropsychological Screen for Mild Cognitive Impairment
CANTAB: Cambridge Neuropsychological Test Automated Battery
CNS Vital Signs: Central Nervous System Vital Signs
COGDRAS-D: Cognitive Drug Research Computerized Assessment System for Dementia
MicroCog: MicroCog: Assessment of Cognitive Functioning
SA-SU: self-administered version first, supervised version second
SU-SA: supervised version first, self-administered version second
