# Supplementary material for: Comparison Between a Self-Administered and Supervised Version of a Web-Based Cognitive Test Battery: Results From the NutriNet-Santé Cohort Study
Source: J Med Internet Res. 2016 Apr 5;18(4):e68. doi: 10.2196/jmir.4862 (PMC4837293; doi:10.2196/jmir.4862)
Supplement: Multimedia Appendix 1 [file jmir_v18i4e68_app1.pdf]

Multimedia Appendix 1. Performance on cognitive tests according to version

Test[a]	Variable[b]	Self-administered version		Supervised version	
		p50	p25; p75	p50	p25; p75
Click	Mean time (s)	4.12	3.51; 5.01	4.49	3.83; 5.64
Maze A	Mean time (s)	108.94	80.97; 148.23	111.52	85.79; 145.95
	Mean nb of clicks (n)	57.67	50.33; 70.00	56.33	50.33; 62.00
	Mean nb of total errors (n)	16.67	11.67; 26.33	14.67	11.33; 18.67
Maze B	Time (s)	72.95	51.24; 98.20	81.83	63.58; 111.25
	Nb of clicks (n)	47.00	39.00; 54.00	45.00	41.00; 53.00
	Nb of total errors (n)	10.00	5.00; 15.00	9.00	6.00; 14.00
Cards	Composite variable[c]	1.26	1.14; 1.37	1.26	1.10; 1.38
	Nb of correct answers (n)	30.00	30.00; 30.00	30.00	30.00; 30.00
	Time (s)	79.36	72.85; 87.74	79.38	72.52; 90.83
Marbles	Composite variable[c]	2.67	1.76; 3.51	2.10	1.44; 3.02
	Nb of incorrect answers (n)	5.00	2.00; 11.00	5.00	2.00; 10.00
	Time (s)	37.42	28.38; 55.93	47.24	32.93; 69.08

Abbreviations: nb, number; p, percentile.

[a] Maze A: Sum of the initial three rounds of the Maze task. Maze B: Final (fourth) round of the Maze task.

[b] Lower scores indicate better performances, except for the Cards- and Marbles- composite variables, where higher scores indicate better performances.

[c] ([1/ (incorrect answers+1000)] /time) * 100,000.
