# Supplementary material for: Comparison Between a Self-Administered and Supervised Version of a Web-Based Cognitive Test Battery: Results From the NutriNet-Santé Cohort Study
Source: J Med Internet Res. 2016 Apr 5;18(4):e68. doi: 10.2196/jmir.4862 (PMC4837293; doi:10.2196/jmir.4862)
Supplement: Multimedia Appendix 2 [file jmir_v18i4e68_app2.pdf]

Multimedia Appendix 2. Spearman correlations between the different cognitive test variables for the self-administered version of the cognitive test battery NutriCog, n=189[a]

	Click: Mean time	Maze A[b]: Mean time	Maze A[b]: Mean clicks	Maze A[b]: Mean total errors	Cards: Composite variable	Marbles: Composite variable	Maze B[b]: Time	Maze B[b]: Clicks	Maze B[b]: Total errors
**Click: Mean time**	*1.00*	0.47	0.28	0.29	-0.49	-0.51	0.50	0.31	0.31
**Maze A[b]: Mean time**	0.47	*1.00*	0.80	0.82	-0.54	-0.38	0.78	0.59	0.57
**Maze A[b]: Mean clicks**	0.28	0.80	*1.00*	0.99	-0.36	-0.33	0.71	0.75	0.72
**Maze A[b]: Mean total errors**	0.29	0.82	0.99	*1.00*	-0.38	-0.33	0.70	0.72	0.70
**Cards: Composite variable[c]**	-0.49	-0.54	-0.36	-0.38	*1.00*	0.45	-0.49	-0.32	-0.32
**Marbles: Composite variable[c]**	-0.51	-0.38	-0.33	-0.33	0.45	*1.00*	-0.47	-0.39	-0.39
**Maze B[b]: Time**	0.50	0.78	0.71	0.70	-0.49	-0.47	*1.00*	0.84	0.83
**Maze B[b]: Clicks**	0.31	0.59	0.75	0.72	-0.32	-0.39	0.84	*1.00*	0.98
**Maze B[b]: Total errors**	0.31	0.57	0.72	0.70	-0.32	-0.39	0.83	0.98	*1.00*

[a] The p-values for all listed Spearman correlation coefficients were <0.0001.

[b] Maze A: Sum of the initial three rounds of the Maze task. Maze B: Final (fourth) round of the Maze task.

[c] ([1/ (incorrect answers+1000)] /time) * 100,000.
