# Supplementary material for: Comparison Between a Self-Administered and Supervised Version of a Web-Based Cognitive Test Battery: Results From the NutriNet-Santé Cohort Study
Source: J Med Internet Res. 2016 Apr 5;18(4):e68. doi: 10.2196/jmir.4862 (PMC4837293; doi:10.2196/jmir.4862)
Supplement: Multimedia Appendix 3 [file jmir_v18i4e68_app3.pdf]

Multimedia Appendix 3. Concordance of cognitive tests performances according to version: Spearman correlation coefficients, stratified by participant characteristics (n=189)[a]

Test[b]	Variable[c]	<50 y	≥50 y	Low[c] education	High[c] education	Men	Women	Web novice	Web expert
Click	Mean time (s)	0.59	0.63	0.80	0.69	0.79	0.69	0.59	0.72
Maze A	Mean time (s)	0.44	0.40	0.35	0.65	0.54	0.59	0.61	0.54
	Mean clicks (n)	0.24	0.37	0.25	0.44	0.46	0.42	0.30	0.44
	Mean total errors (n)	0.21	0.34	0.23	0.47	0.49	0.42	0.34	0.43
Maze B	Time (s)	0.51	0.37	0.45	0.54	0.51	0.52	0.41	0.51
	Clicks (n)	0.45	0.33	0.32	0.44	0.51	0.36	0.34	0.45
	Mean total errors (n)	0.45	0.30	0.33	0.44	0.51	0.36	0.27	0.44
Cards	Composite variable[d]	0.52	0.68	0.52	0.69	0.64	0.65	0.49	0.63
Marbles	Composite variable[d]	0.43	0.40	0.39	0.56	0.47	0.54	0.41	0.51

[a] Versions of the cognitive test battery: web-based version and supervised version. Frequencies (n) per subgroup: <50 y: 62; ≥50 y: 127; low education: 67; high education: 122; men: 72; women: 117; web novice: 19; web expert: 166.

[b] Maze A: Sum of the initial three rounds of the Maze task. Maze B: Final (fourth) round of the Maze task.

[c] Low education: less than two years of education after high-school. High education: At least two years of education after high-school.

[d] ([1/ (incorrect answers+1000)] /time) * 100,000.
